# Emergency endovascular treatment using a Viabahn stent graft for upper and lower extremity arterial bleeding: a retrospective study

**DOI:** 10.1186/s42155-021-00273-z

**Published:** 2021-12-09

**Authors:** Tatsuo Ueda, Satoru Murata, Hiroyuki Tajima, Hidemasa Saito, Daisuke Yasui, Fumie Sugihara, Shohei Mizushima, Takahiko Mine, Hiroshi Kawamata, Hiromitsu Hayashi, Shin-Ichiro Kumita

**Affiliations:** 1 Department of Radiology, Nippon Medical School Hospital, 1-1-5 Sendagi, Bunkyo-ku, 113-8603 Tokyo, Japan; 2 Center for Interventional Radiology, Teikyo University Chiba Medical Center, 3426-3 Anesaki, 299-0011 Ichihara City, Chiba Japan; 3 Department of Diagnostic Radiology, Saitama Medical University International Medical Center, 1397-1 Yamane, Saitama 350-1298 Hidaka City, Japan; 4 Department of Radiology, Nippon Medical School Musashi Kosugi Hospital, 1-396 Kosugi-machi, Nakahara-ku, 211-8533 Kawasaki City, Kanagawa Japan; 5 Department of Radiology, Nippon Medical School Chiba Hokusoh Hospital, 1715 Kamagari, Chiba 270-1694 Inzai City, Japan; 6 Department of Radiology, Yokohama Rosai Hospital, 3211 Kozukue-Cho, Kohoku-Ku, 222-0036 Yokohama City, Kanagawa Japan

**Keywords:** Viabahn, Stent graft, Upper extremity, Lower extremity, Arterial bleeding

## Abstract

**Background:**

A Viabahn stent graft (SG) is a heparin-coated self-expandable SG for lower extremity arterial disease that exhibits high flexibility and accuracy in the delivery system. This study aimed to evaluate the short-term efficacy and safety of emergency endovascular treatment (EVT) using a Viabahn SG for upper and lower extremity arterial bleeding (ULEAB).

**Methods:**

Consecutive patients with ULEAB who underwent emergency EVT using the Viabahn SG between January 2017 and August 2021 were retrospectively reviewed. The indications for EVT, location of artery, technical success, clinical success, limb ischemia, periprocedural complications, bleeding-related mortality, 30-day mortality, diameter of the target artery, diameter of the SG, neck length, rebleeding, endoleaks, and patency of the SGs at 1, 3, 6, and 12 months were evaluated.

**Results:**

EVT using the Viabahn SG was performed in 22 patients (mean age, 72.0 ± 13.0 years; 11 men) and 23 arteries (upper, 6; lower, 17). The indications for EVT were pseudoaneurysm (*n* = 13, 59.1%), extravasation (*n* = 9, 39.1%), and inadvertent arterial cannulation (*n* = 1, 4.3%). The anatomical locations of the 23 ULEAB injuries were the brachiocephalic (1 [4.3%]), subclavian (3 [13.0%]), axillary (1 [4.3%]), brachial (1 [4.3%]), common iliac (4 [17.4%]), external iliac (8 [34.8%]), common femoral (2 [8.7%]), superficial femoral (2 [8.7%]), and popliteal (1 [4.3%]) arteries. The technical and clinical success rates were 100%. The rates of limb ischemia, periprocedural complications, and bleeding-related mortality were 0%, whereas the 30-day mortality rate was 22.7%. The mean diameters of the arteries and SGs were 7.7 ± 2.2 and 8.9 ± 2.3 mm, respectively. The mean neck length was 20.4 ± 11.3 mm. No endoleaks or rebleeding occurred during the follow-up period (mean, 169 ± 177 days). Two SG occlusions without limb ischemia occurred in the external iliac and brachial arteries after 1 and 4 months, respectively. Subsequently, cumulative SG patency was confirmed after 1, 3, 6, and 12 months in 91.7%, 91.7%, 81.5%, and 81.5% of patients, respectively.

**Conclusions:**

Emergency EVT using the Viabahn SG for ULEAB was effective and safe according to short-term outcomes. Appropriate size selection and neck length are important for successful treatment. SG patency was good after 1, 3, 6, and 12 months.

## Background

Conventionally, upper and lower extremity arterial bleeding (ULEAB) has been treated surgically; however, currently, a less invasive endovascular approach is favored (Waes et al. 2013; Fox et al. 2012; Katsanos et al. 2009). Endovascular treatment (EVT) with a stent graft (SG) is ideal for ULEAB because it simultaneously allows for hemostasis and maintains the blood flow of peripheral limb arteries (Katsanos et al. 2009; Ueda et al. 2017, 2021; Venturini et al. 2017; Ormiston et al. 2020). A Viabahn SG (W. L. Gore & Associates, Flagstaff, AZ, USA) is a heparin-coated self-expandable SG for lower extremity arterial disease that exhibits high flexibility and accuracy in the delivery system. Compared with a balloon-expandable SG, this high flexibility is more suitable for endovascular repair of tortuous arteries or highly mobile sites, such as the axillary, iliac, common femoral, and popliteal arteries. Therefore, EVT with Viabahn SGs may be one of the best treatment options for ULEABs. Nevertheless, to date, reports regarding the outcomes of EVT with Viabahn SGs for ULEAB are limited (Ormiston et al. 2020; DuBose et al. 2012; Kufner et al. 2015; Backer et al. 2015; Desai et al. 2014). This study aimed to evaluate emergency EVT using a Viabahn SG for ULEAB at various locations of arteries, focusing on technical aspects, short-term efficacy, and safety.

## Methods

### Patients

This study is a retrospective multicenter series. This study included consecutive patients who underwent emergency EVT using the Viabahn SG for ULEAB between January 2017 and August 2021. The eligibility criteria were as follows: (1) evidence of ULEABs on computed tomography angiography (CTA), (2) diameter of the target vessel between 4 and 12 mm, and (3) no contraindications to heparinization or contrast media. We did not consider a surgical approach because EVT using the Viabahn SG is less invasive and can be performed more quickly than surgical repair. Particularly, surgical repair under general anesthesia was considered a high-risk procedure for hemodynamically unstable patients with massive hematoma. This study was approved by the institutional review board of all hospitals, and informed consent was obtained from all patients before treatment.

### Endovascular procedure

EVT was performed through a common femoral, brachial, axillary, or radial artery access with a 4-Fr sheath (Supersheath; Medikit, Tokyo, Japan) under local anesthesia with conscious sedation or general anesthesia. A 4-Fr catheter (GLIDECATH; Terumo, Tokyo, Japan) was advanced to the distal side of the target artery with the aid of a 0.035-inch guidewire (Radifocus Guide Wire M; Terumo, Tokyo, Japan). The guidewire was exchanged for a 0.035-inch stiff wire (Amplatz Super Stiff^TM^; Boston Scientific, Natick, MA, USA) to exchange the sheath for a 6–12-Fr sheath (Medikit) or 6-Fr guiding sheath (Destination; Terumo, Tokyo, Japan). The sheath or guiding sheath was then advanced to the distal side of the target artery or as close to the target artery as possible. A 0.035-inch stiff wire or 0.018-inch stiff wire (V-18^TM^; Boston Scientific, Natick, MA, USA) was used for SG delivery. The target artery was measured by either arteriography or pre-treatment CTA imaging. Considering that potential vasospasm or shock may affect the artery diameter, CTA imaging of the pre-hemorrhage state was also used for the measurement. The SG diameter was approximately 10% oversized to the diameter of the target artery. The SG size selection was based on our previous experience. The SG length covered the entire target artery. The Viabahn SG (diameter, 5–13 mm; length, 50 or 100 mm) was advanced until it covered the entire site of the target artery and was then deployed. SG deployment was performed without a prolonged balloon-inflated technique. Anticoagulation therapy was not performed during the procedure. Antibiotics were administered during the procedure in infectious patients. In the case of a branch artery >2 mm in diameter within 5 mm of the target artery, embolization of the branch artery was performed with coils (Tornado; Cook Medical, Bloomington, IN, USA; Interlock; Boston Scientific, Natick, MA, USA) or an AMPLATZER™ Vascular Plug 2 (AVP 2; St. Jude Medical, St. Paul, MN, USA) to avoid a type 2 endoleak (EL). Angiography was immediately performed after the deployment without post-dilatation of the SG. If the angiogram showed type 1 or 3 EL, an additional percutaneous transluminal angioplasty with the same SG diameter and/or an additional SG placement that partially overlapped the first SG was performed to treat the EL. After the procedure, if there were no contraindications, anticoagulation therapy based on dual antiplatelet therapy (aspirin 100 mg/day and clopidogrel 75 mg/day) was prescribed for at least 6 months to prevent SG thrombosis, and then, single antiplatelet therapy (aspirin 100 mg/day or clopidogrel 75 mg/day) was maintained for lifetime. In the case of hemorrhagic patients, dual antiplatelet therapy was started after the patient’s vital sign was stable. Follow-up CTA was performed at 1, 3, 6, and 12 months after treatment.

### Assessment of EVT efficacy

The following data were collected: enrolled patient number, age, sex, cause, indication, location, approach site, diameter of the target artery, diameter of the SG, oversizing of the SG, length of the SG, neck length, branch artery embolization, procedure time, anticoagulation therapy, and CTA follow-up duration. Neck length was defined as the minimum distance between the edge of the SG and the injured or bleeding point. The technical success, clinical success, limb ischemia, periprocedural complications, bleeding-related mortality, 30-day mortality, rebleeding, ELs, and SG patency were assessed at 1, 3, 6, and 12 months. Technical success was defined as the disappearance of the pseudoaneurysm and extravasation with preserved blood flow in the limb artery on the angiogram. Clinical success was defined as complete hemostasis within 30 days of the procedure. Limb ischemia was defined as ischemic sequelae of the limb of the target artery. Complications were defined as major complications that required therapy, according to the Society of Interventional Radiology classification (Khalilzadeh et al. 2017). Rebleeding, ELs, and SG patency were evaluated using CTA images.

### Statistical analyses

Continuous variables are presented as mean ± standard deviation, whereas categorical data are presented as percentages. Normality testing was employed to determine if data were normally distributed. Kaplan–Meier analyses were performed for SG patency using the Statistical Package for the Social Sciences version 21 (IBM Corp., Armonk, NY, USA).

## Results

### Patient characteristics

**Fig. 1:**
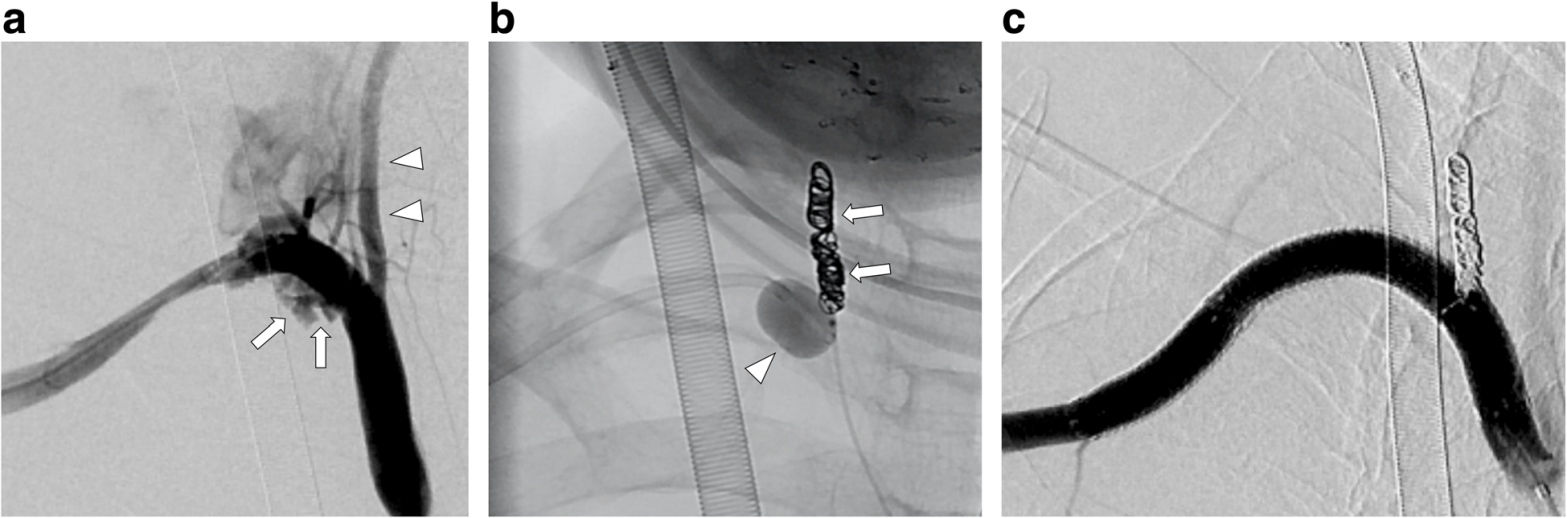
A 64-year-old woman with right subclavian artery injury by an inadvertent puncture. **A** Pre-treatment angiogram shows extravasation from the proximal site of the right subclavian artery (arrows). The right vertebral artery branches close to the extravasation (arrowheads). **B** After confirming that the contralateral vertebral artery is patent and joins the ipsilateral vertebral artery, the right vertebral artery is embolized by coils (arrows) to avoid type 2 endoleaks under balloon occlusion (arrowhead) of the right subclavian artery. **C** Angiogram after endovascular therapy with Viabahn stent grafts (8 mm × 5 cm and 8 mm × 10 cm) shows the disappearance of the extravasation without endoleak

**Fig. 2:**
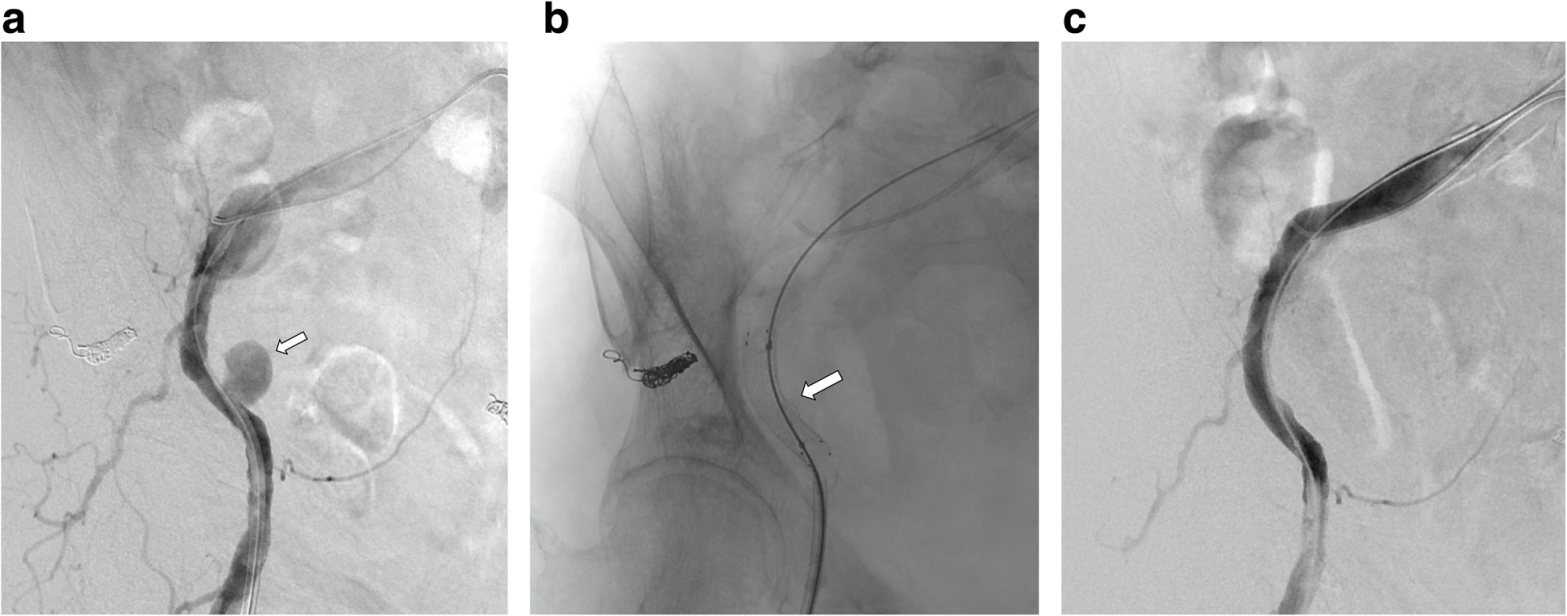
A 76-year-old woman with right external iliac artery pseudoaneurysm by uterine carcinoma. **A** Pre-treatment angiogram shows a pseudoaneurysm from the distal site of the right external iliac artery (arrow). **B** The Viabahn stent graft (9 mm × 5 cm) (arrowhead) is placed at the external iliac artery. The coils in the internal iliac artery were used for the previous treatment of uterine carcinoma. **C** Angiogram after endovascular therapy with a Viabahn stent graft shows disappearance of the pseudoaneurysm without endoleak

A summary of the results is provided in Table 1. The study included 22 patients (mean age, 72.0 ± 13.0 years; 11 men) with 23 ULEAB injuries, as one patient had two ULEAB injuries. ULEAB injuries were noted in the arteries, including the brachiocephalic (*n* = 1), subclavian (*n* = 3) (Fig. 1), axillary (*n* = 1), brachial (*n* = 1), common iliac (*n* = 4), external iliac (*n* = 8) (Fig. 2), common femoral (*n* = 2), superficial femoral (*n* = 2), and popliteal (*n* = 1) arteries. The brachial and common femoral arteries are generally treated surgically. However, all patients with injuries involving these arteries were hemodynamically unstable and exhibited massive hematoma, and surgical repair under general anesthesia was considered a high-risk procedure. Therefore, we decided to treat the cases with EVT. The causes of ULEAB were post-endovascular therapy (*n* = 9), postoperative complications (*n* = 3), idiopathic causes (*n *= 3), infection (*n* = 2), trauma (*n* = 2), carcinoma (*n* = 2), and postradiation therapy (*n* = 1). The indications for EVT included active uncontained hemorrhage (9), contained hemorrhage resulting in pseudoaneurysm formation (13), and iatrogenic arterial injury (1).

**Table 1:** Summary of results

Factor		
Patients		22
Cases		23
Age (years, mean ± SD)		72.0 ± 13.0 (range, 36–90)
Sex (male/female)		11/11
Indication (pseudoaneurysm/extravasation/inadvertent arterial cannulation)		13/9/1
Location of the artery (BCA/SCA/AXA/BA/CIA/EIA/CFA/SFA/POP)		1/3/1/1/4/8/2/2/1
Approach site (femoral/axillary/brachial/radial)		20/1/1/1
Artery diameter (mm, mean ± SD)		7.7 ± 2.2 (range, 5–12)
Stent graft diameter (mm, mean ± SD)		8.9 ± 2.3 (range, 6–13)
Stent graft oversizing (%, mean ± SD)		16.1 ± 7.8 (range, 0–33)
Stent graft length (5/10 cm)		20/8
Neck length (mm, mean ± SD)		20.4 ± 11.3 (range, 3–50)
Branch artery embolization		3
Procedure time (min, mean ± SD)		35.5 ± 21.2 (range, 8–86)
Technical success (%)		100
Clinical success (%)		100
Limb ischemia (%)		0
Periprocedural complications (%)		0
Bleeding-related mortality (%)		0
30-day mortality (%)		22.7
Anticoagulation therapy (%)		77.3
CTA follow-up duration (days, mean ± SD)		168.8 ± 176.5 (range, 3–655)
Rebleeding (%)		0
Endoleaks (%)		0
Stent graft patency (%)	1 month	91.7
	3 months	91.7
	6 months	81.5
	12 months	81.5

*AXA, axillary artery; BA, brachial artery; BCA, brachiocephalic artery; CFA, common femoral artery; CIA, common iliac artery; CTA, computed tomography angiography; EIA, external iliac artery; POP, popliteal artery; SCA, subclavian artery; SFA, superficial femoral artery; SD, standard deviation*

### Procedure results

EVT was performed through the common femoral artery in 20 patients, brachial artery in 1 patient, axillary artery in 1 patient, and radial artery in 1 patient. The brachial artery approach was used in cases where it was impossible to approach the common femoral artery, the axillary artery approach was used in cases where it was impossible to approach the common femoral artery and a 12-Fr sheath was required, and the radial artery approach was used in the treatment of brachial artery pseudoaneurysm. The axillary artery approach was performed using the surgical cut-down. The other approaches were performed percutaneously and hemostasis of the access site was secured by manual compression or using a Perclose ProGlide suture-mediated closure system (Abbott Vascular, Santa Clara, CA, USA). There were no complications related to hemostasis in the access site. The mean diameters of the arteries and SGs were 7.7 ± 2.2 (range, 5–12) and 8.9 ± 2.3 (range, 6–13) mm, respectively. The 50-mm SG was selected for 20 patients, and the 100-mm SG was selected for 8 patients. The mean neck length was 20.4 ± 11.3 (range, 3–50) mm in all patients. Branch artery embolization was performed in three patients (one vertebral artery case and two internal iliac artery cases) (Fig. 1). The mean procedure time was 35.5 ± 21.2 (range, 8–86) min.

### Initial and midterm results

**Fig. 3:**
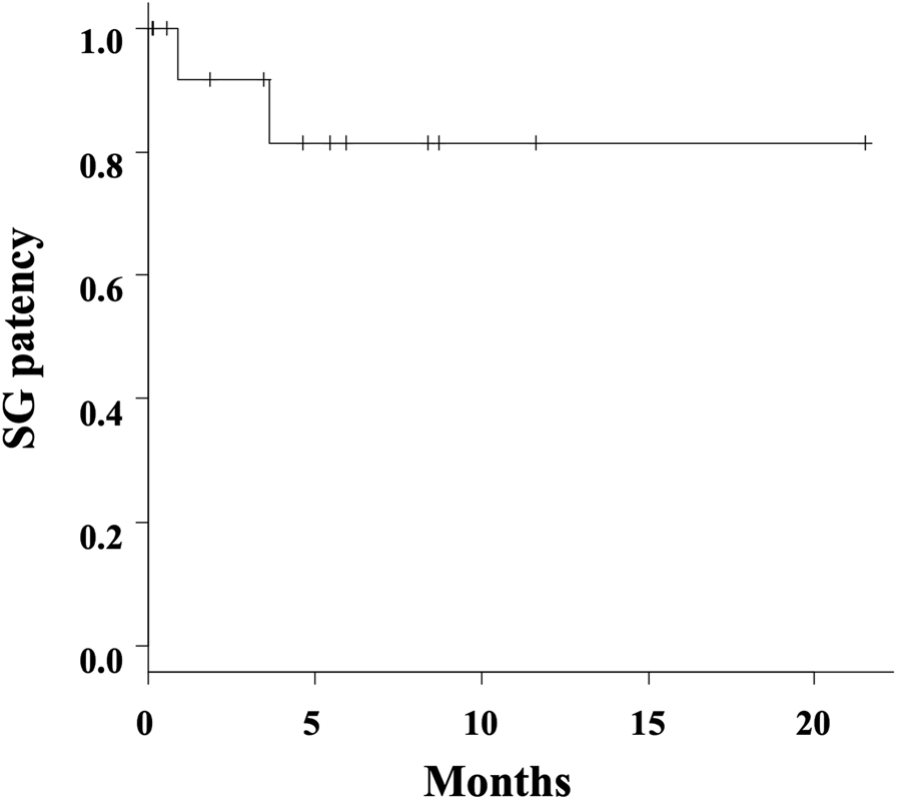
Stent graft patency. A Kaplan–Meier curve reveals stent graft patency over time for the Viabahn stent grafts. The transverse axis shows the time (months) after the procedure. SG, stent graft

Technical and clinical success was achieved in all patients. Ischemia of the limbs and periprocedural complications were both at 0%. Five patients died 3, 6, 13, 24, and 29 days after SG treatment because of acute myocardial infarction in two, carcinoma in two, and bowel ischemia in one patient, respectively. Therefore, the 30-day mortality rate was 22.7% (5/22). However, there were no bleeding-related deaths. Anticoagulation therapy was prescribed to 17 of the 22 patients (77.3%). CTA postoperative follow-ups were performed on 15 of the 22 patients (68.2%), and the mean CTA follow-up period was 168.8 ± 176.5 (range, 3–655) days. No rebleeding or EL was observed during the CTA follow-up period. Two SG occlusions without limb ischemia occurred in the external iliac artery and brachial artery after 1 and 4 months, respectively. Subsequently, cumulative SG patency was confirmed after 1, 3, 6, and 12 months in 91.7%, 91.7%, 81.5%, and 81.5% of patients, respectively (Fig. 3). The patient with SG occlusion in the brachial artery only received aspirin when the SG occlusion occurred, and the target artery and SG diameters were 6 and 8 mm, respectively. The other occluded patient in the external iliac artery had iliac occlusion disease prior to the SG treatment. The SG lengths were 50 (external iliac artery case) and 100 (brachial artery case) mm, respectively. We did not treat the SG occlusions because both patients were asymptomatic. There were no SG fracture cases including the mobile sites such as the axillary, common femoral, and popliteal arteries during the follow-up.

## Discussion

The technical and clinical success rates were consistent with those in previous studies, at 97−100% (DuBose et al. 2012; Kufner et al. 2015; Backer et al. 2015; Desai et al. 2014). Appropriate diameter size selection is important to avoid rebleeding and type 1 ELs (T1ELs). Since the most important goal in treating ULEAB is hemostasis, avoiding an undersized diameter is crucial. In patients with hypovolemic shock, the artery diameter could become smaller because of vasospasm; therefore, it would be important to oversize the SG further but be mindful of not excessively oversizing it so as not to affect the patency. Neck length is also a critical factor in avoiding rebleeding and T1EL. It should be secured at a minimum of 20 mm, according to the manufacturer’s instructions. We used a mean neck length of 20.4 (range, 3–50) mm without observing any T1EL. These results suggest that it is possible to treat ULEAB without T1EL if the diameter size selection and neck length are appropriate. Although a balloon-expandable SG enables more accurate deployment, a self-expandable SG, more flexible and adaptable, is more suitable in tortuous arteries, such as the limb arteries. Furthermore, a balloon-expandable SG has stronger radial force than a self-expandable SG, and it may cause additional damage to the high fragility of the injured arterial wall. ​Indeed, we did not use a balloon-expandable SG in case 1 (Fig. 1) because it had a potential risk to worsen the injured artery due to excessive radial force. Anticoagulation therapy was not performed during the procedure. In general, although periprocedural anticoagulation therapy is important in SG replacement to avoid thrombus, in our opinion, anticoagulation therapy is not mandatory for bleeding cases. Controlling bleeding is more important than avoiding thrombus; particularly, it is better to reduce the bleeding risk in the hemodynamically unstable cases. In the case of using SGs in patients with mycotic pseudoaneurysms or sepsis, there may be higher risks of rebleeding, ELs, and SG occlusion due to the spread of inflammation to the vessel walls. However, in our opinion, infection or sepsis is not an absolute contraindication for SG placement. In a literature review by Miller et al., no case of SG infection was reported among outcomes (Todd Miller et al. 2007). In the present series including two cases of infection, we did not find any clinical or imaging signs of SG infection during follow-up. We believe that control of infection by antibiotic administration and maintenance of the therapy for a long period, along with the surgical or percutaneous drainage of collections, are crucial to prevent the risk of SG infection. Our SG patency was consistent with those reported in previous studies at 68–100% (DuBose et al. 2012; Kufner et al. 2015; Backer et al. 2015; Desai et al. 2014). It is difficult to identify the cause of occlusion; however, anticoagulation therapy, oversized SG, location, infection, and smaller vessel diameters may be contributing factors. Saxon et al. (2013). Furthermore, Ueda et al. (2021). However, we used a device oversized by >20% (8-mm SG for a 6-mm artery diameter) in an SG occlusion case and did not need to treat the occlusions, as there was no evidence of limb ischemia due to the development of collateral circulation. However, the limb arteries are potentially at risk of ischemia; therefore, SG occlusion should be prevented. The limitations of the current study include its retrospective nature, single-arm design, and limited imaging follow-up data available for only a small number of patients.

In conclusion, emergency EVT using a Viabahn SG for ULEAB was effective and safe for short-term outcomes, with low rates of limb ischemia, periprocedural complications, and bleeding-related mortality. Appropriate size selection and neck length are important for successful treatment. SG patency was good after 1, 3, 6, and 12 months. Long-term follow-up and a larger sample size are suggested for further treatment evaluation.

## Data Availability

Data sharing is not applicable to this article as no datasets were generated or analyzed during the current study.

## Abbreviations

CTA: computed tomography angiography
EL: endoleak
EVT: endovascular treatment
ULEAB: upper and lower extremity arterial bleeding
SG: stent graft
T1EL: type 1 endoleak
